# Data on cytotoxicity of plant essential oils in A549 and Detroit 551 cells

**DOI:** 10.1016/j.dib.2020.106186

**Published:** 2020-08-16

**Authors:** Yeong-Min Yoo, Jae-Hwan Lee, Eui-Man Jung, Mi-Jin Park, Jae-Woo Kim, Jiyoon Yang, Eui-Bae Jeung

**Affiliations:** a Laboratory of Veterinary Biochemistry and Molecular Biology, College of Veterinary Medicine, Chungbuk National University, Chungbuk 28644, Republic of Korea; b Division of wood chemistry & Microbiology, Department of Forest Products, National Institute of Forest Science, Seoul 02455, Republic of Korea

**Keywords:** Plant essential oils, A549 cells, Detroit 551 cells, CCK

## Abstract

To secure the safety for industrial applications of plant essential oils, it is necessary to determine the inhibitory concentration and inhibitory mechanism of cell proliferation in skin cells and lung cells. Considering inhalation through the respiratory system and skin contact of humans with essential oils, we used human lung cancer cells A549 and human skin fibroblasts Detroit 551 cells for all experiments. In this study, we examined IC_50_ values and protein levels of cell cycle markers (cyclin A, cyclin B, cyclin D, and cyclin E) and apoptosis marker (caspase-3) after exposure to 10 plant essential oils, including *Dendranthema indicum* (L.) Des Moul, *Peucedanum japonicum* Thunb, *Dendranthema zawadskii* var. *latilobum* (Maxim.) Kitam, *Agastache rugosa* (Fisch.&Mey.) Kuntze, *Vitex rotundifolia* L.f, *Pinus rigida* Mill*; Orixa japonica* Thunb, *Pinus strobus* L, *Chamaecyparis pisifera* (Siebold et Zucc.) Endl. var. *filifera* Beissn. et Hochst, and *Citrus sunki* Hort. ex Tanaka. After the treatment of A549 and Detroit 551 cells to varying concentrations of the 10 plant essential oils, IC_50_ values were determined by CCK analysis, whereas protein expressions of the four cyclins and caspase-3 were identified by Western blotting analysis. We believe that by examining the degree and mechanism of cell proliferation inhibition exerted by essential oils on skin and lung cells of humans, data obtained in this study can provide guidelines for the industrial application of plant essential oils.

**Specifications Table**

**Table:** 

Subject	*Agricultural and Biological Sciences*
Specific subject area	*Natural products research*
Type of data	*Graphs, Tables and Figures*
How data was acquired	*Graph and* IC_50_*by Graph Pad Prism, Western blotting*
Data format	*The raw data of excel files were analyzed, and* IC_50_*values were obtained.*
Parameters for data collection	*The cell survival curves were achieved by CCK and subsequently analyzed to determine the* IC_50_*of plant essential oils. Protein expressions of four cell cycle markers (cyclin A, cyclin B, cyclin D, and cyclin E) and apoptosis marker (caspase-3) were identified by Western blot analysis.*
Description of data collection	*Cell survival curves were calculated using Excel and Graph Pad Prism, and* IC_50_*values were obtained. Proteins were extracted using RIPA buffer, and resultant proteins were quantitated by Western blot analysis.*
Data source location	*Chungbuk National University, Chungbuk, 28,644 Republic of Korea*
Data accessibility	*All data are provided with this article, and raw data are available in this article as supplementary files.*

**Value of the data**

• This data provides guidelines and safety for industrial applications of plant essential oils.
• This data may be useful to understand the IC_50_ values of plant essential oils in A549 and Detroit 551 cells.
• This data could be actively used for determining molecular mechanisms of the cell cycle and apoptosis of plant essential oils in the A549 and Detroit 551 cells.
• The data obtained can support researchers in the field of molecular mechanisms of plant essential oils, and individuals who work with plants.

## 1 Data

Essential oils are secondary metabolites produced by plants that are highly aromatic and are a mixture of various components. Essential oils contain numerous monoterpenes and sesquiterpenes and can be obtained from the flowers, leaves, stems, and roots of plants [1]. Essential oils have a variety of applications in several areas, such as preservatives, antioxidants, and perfumes [2,3]. Considering the wide applications, we examined cytotoxicity and protein marker data in A549 and Detroit 551 cells.

**Fig. 1:**
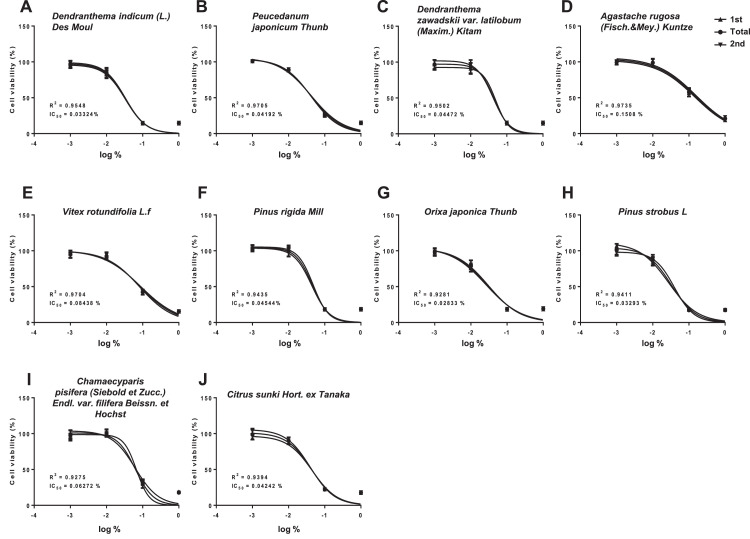
Cell survival curves for determining the IC_50_ values of essential oils in the A549 cell. OD values were measured after subjecting cells with essential oils to the CCK-8 assay. Cell viability was determined by applying the formula OD_sample_ / OD_control_ × 100 (%), and the IC_50_ values were determined through the obtained survival curve. (A) *Dendranthema indicum* (L.) Des Moul, (B) *Peucedanum japonicum* Thunb, (C) Dendranthema zawadskii var. *latilobum* (Maxim.) Kitam, (D) *Agastache rugosa* (Fisch.&Mey.) Kuntze, (E) Vitex rotundifolia L.f, (F) *Pinus rigida Mill*, (G) *Orixa japonica* Thunb, (H) *Pinus strobus* L, (I) *Chamaecyparis pisifera* (Siebold et Zucc.) Endl. var. *filifera* Beissn. et Hochst, and (J) *Citrus sunki* Hort. ex Tanaka.

CCK-8 analysis was performed to obtain cell survival and cytotoxicity IC_50_ values of 10 essential oils in A549 cells (Fig. 1, Table 1). The IC_50_ value (%) of *A. rugosa* (Fisch. & Mey.) Kuntze (0.1508) was relatively higher, but with lesser toxicity, than values obtained for other essential oils (0.02833∼0.08438). *D. indicum* (L.) Des Moul, *P. japonicum* Thunb, *O. japonica* Thunb, *P. strobus* L, and *C. sunki* Hort. ex Tanaka induced toxicity at 0.01%.

**Table 1:** The IC_50_ values of essential oils in the A549 cell line.

Essential oils	IC_50_ (%, v/v)
*Dendranthema indicum* (L.) Des Moul	0.03324
*Peucedanum japonicum* Thunb	0.04192
*Dendranthema zawadskii* var. *latilobum* (Maxim.) Kitam	0.04472
*Agastache rugosa* (Fisch.&Mey.) Kuntze	0.1508
*Vitex rotundifolia* L.f	0.08438
*Pinus rigida* Mill	0.04544
*Orixa japonica* Thunb	0.02833
*Pinus strobus* L	0.03293
*Chamaecyparis pisifera* (Siebold et Zucc.) Endl. var. *filifera* Beissn. et Hochst	0.06272
*Citrus sunki* Hort. ex Tanaka	0.04242

**Fig. 2:**
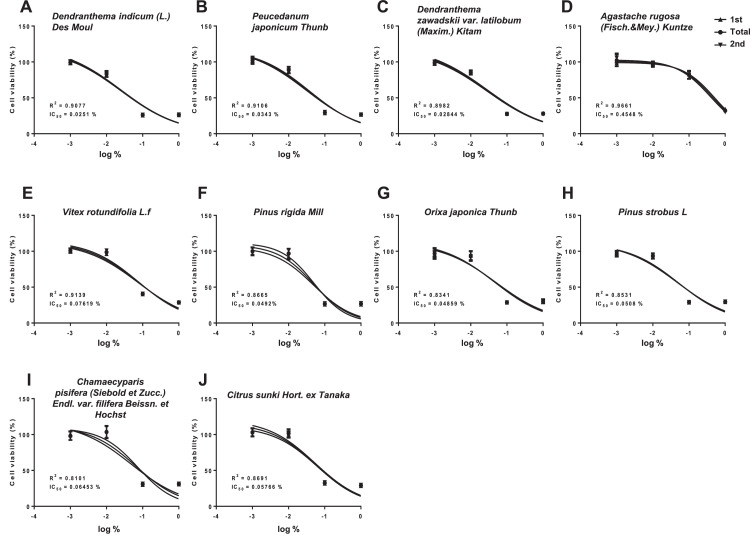
Cell survival curves for determining the IC50 values of essential oils in the Detroit 551 cell. After exposing the cells to varying concentrations of essential oils, the OD values were obtained by subjecting the treated cells to the CCK-8 assay. Cell viability was determined by applying the formula OD_sample_ / OD_control_ × 100 (%), and the IC_50_ values were determined through the obtained survival curve. (A) *D. indicum* (L.) Des Moul, (B) *P. japonicum* Thunb, (C) *D. zawadskii* var. *latilobum* (Maxim.) Kitam, (D) *A. rugosa* (Fisch.&Mey.) Kuntze, (E) *V. rotundifolia* L.f, (F) *P. rigida Mill*, (G) *O. japonica* Thunb, (H) *P. strobus* L, (I) *C. pisifera* (Siebold et Zucc.) Endl. var. *filifera* Beissn. et Hochst, and (J) *C. sunki* Hort. ex Tanaka.

The cell survival and cytotoxicity IC_50_ values of 10 essential oils were also examined in Detroit 551 cells using CCK-8 analysis (Fig. 2, Table 2). The IC_50_ value obtained with: *D. indicum* (L.) Des Moul 0.0251%; *P. japonicum* Thunb 0.0343%; *D. zawadskii* var. *latilobum* (Maxim.) Kitam 0.02844%; *A. rugosa* (Fisch.&Mey.) Kuntze 0.4548%; *V. rotundifolia* L.f 0.07619%; *P. rigida* Mill 0.0492%; *O. japonica* Thunb 0.04859%; *P. strobus* L 0.0508%; *C. pisifera* (Siebold et Zucc.) Endl. var. *filifera* Beissn. et Hochst 0.06453%; and *C. sunki* Hort. ex Tanaka 0.05766%.

**Table 2:** The IC_50_ values of essential oils in the Detroit 551 cell line.

Essential oils	IC_50_ (%, v/v)
*Dendranthema indicum* (L.) Des Moul	0.0251
*Peucedanum japonicum* Thunb	0.0343
*Dendranthema zawadskii* var. *latilobum* (Maxim.) Kitam	0.02844
*Agastache rugosa* (Fisch.&Mey.) Kuntze	0.4548
*Vitex rotundifolia* L.f	0.07619
*Pinus rigida* Mill	0.0492
*Orixa japonica* Thunb	0.04859
*Pinus strobus* L	0.0508
*Chamaecyparis pisifera* (Siebold et Zucc.) Endl. var. *filifera* Beissn. et Hochst	0.06453
*Citrus sunki* Hort. ex Tanaka	0.05766

**Fig. 3:**
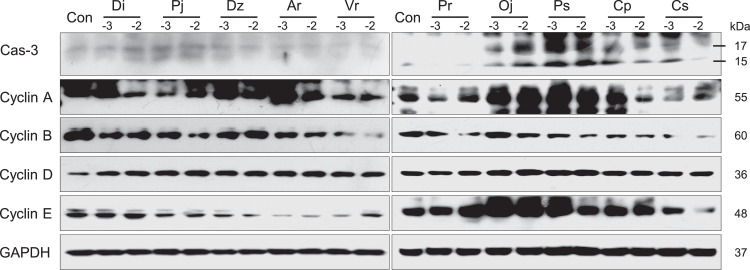
The expression levels of caspase-3, cyclin A, cyclin B, cyclin D, and cyclin E proteins in A549 cells. The protein expression levels were determined by western blot analysis after treating A549 cells with two concentrations of essential oil. The two concentrations used (0.001% and 0.01%) are indicated as −3 and −2, respectively. (Di) *D. indicum* (L.) Des Moul, (Pj) *P. japonicum* Thunb, (Dz) *D. zawadskii* var. *latilobum* (Maxim.) Kitam, (Ar) *A. rugosa* (Fisch.&Mey.) Kuntze, (Vr) *V. rotundifolia* L.f, (Pr) *P. rigida Mill*, (Oj) *O. japonica* Thunb, (Ps) *P. strobus* L, (Cp) *C. pisifera* (Siebold et Zucc.) Endl. var. *filifera* Beissn. et Hochst, and (Cs) *C. sunki* Hort. ex Tanaka. Cas-3, caspase-3.

**Fig. 4:**
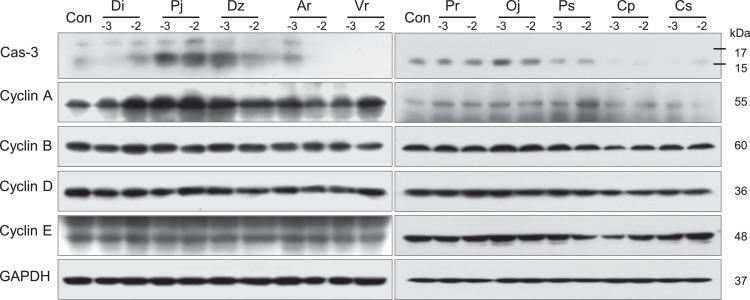
Expression levels of caspase-3, cyclin A, cyclin B, cyclin D, and cyclin E proteins in Detroit 551 cells. The protein expression levels were determined by western blot analysis after treating Detroit 551 cells with two concentrations of essential oil. The two concentrations used (0.001% and 0.01%) are indicated as −3 and −2, respectively. (Di) *D. indicum* (L.) Des Moul, (Pj) *P. japonicum* Thunb, (Dz) *D. zawadskii* var. *latilobum* (Maxim.) Kitam, (Ar) *A. rugosa* (Fisch.&Mey.) Kuntze, (Vr) *V. rotundifolia* L.f, (Pr) *P. rigida Mill*, (Oj) *O. japonica* Thunb, (Ps) *P. strobus* L, (Cp) *C. pisifera* (Siebold et Zucc.) Endl. var. *filifera* Beissn. et Hochst, and (Cs) *C. sunki* Hort. ex Tanaka. Cas-3, caspase-3.

The protein expression levels of cyclin A, cyclin B, cyclin D, and cyclin E in the A549 and Detroit 551 cell lines after exposure to the essential oils are presented in Fig. 3, Fig. 4. In both A549 and Detroit 551 cells, the protein expression levels of cyclin A, cyclin B, cyclin D, and cyclin E were altered after treatment with plant essential oils. In A549 cells, the expression of caspase-3 protein, a key enzyme of apoptosis, was increased in *D. indicum* (L.) Des Moul, *P. japonicum* Thunb, *D. zawadskii* var. *latilobum* (Maxim.) Kitam, *O. japonica* Thunb, *P. strobus* L, and *C. pisifera* (Siebold et Zucc.) Endl. var. *filifera* Beissn. et Hochst. A similar increase was observed in Detroit 551 cells exposed to *D. indicum* (L.) Des Moul, *P. japonicum* Thunb, *D. zawadskii* var. *latilobum* (Maxim.) Kitam, *A. rugosa* (Fisch.&Mey.) Kuntze, *O. japonica* Thunb, and *P. strobus* L.

## 2 Experimental design, materials and methods

### 2.1 Plants essential oils

The following 10 species of plant essential oils were received from the National Institute of Forest Science (Pocheon city, Gyeonggi-do, Republic of Korea): *D. indicum* (L.) Des Moul; *P. japonicum* Thunb; *D. zawadskii* var. *latilobum* (Maxim.) Kitam; *A. rugosa* (Fisch.&Mey.) Kuntze; *V. rotundifolia* L.f; *P. rigida* Mill; *O. japonica* Thunb; *P. strobus* L; *C. pisifera* (Siebold et Zucc.) Endl. var. *filifera Beissn*. et Hochst; *C. sunki* Hort. ex Tanaka.

### 2.2 Cell culture

All cell culture assays were performed as per the protocols suggested by Ahn [4]. Human lung cancer cells A549 and human skin fibroblasts Detroit 551 cells were purchased from Korea Cell Line Bank (KCLB, Seoul, Republic of Korea). Cells were cultured in DMEM high-glucose media (Biowest, France) supplemented with 5% penicillin-streptomycin solution (Biowest, France) and 10% fetal bovine serum (FBS) at 37 °C in a 5% CO_2_ humidified culture incubator (Sanyo, Japan).

### 2.3 Cell proliferation assay

To determine cell survival after exposure to essential oils, 4000 cells per well were seeded in a 96-well plate and incubated for 24 h. Fresh medium was added to the cultured cell plates, and cells were subsequently treated to varying concentrations of essential oils. The plant essential oils were diluted to 0.001%, 0.01%, 0.1%, and 1% concentrations with 0.1% DMSO-plus 5% DMEM media (specimen volume/media volume). After further incubation 24 h, plant essential oils and media were removed, and treated cells were washed twice with DPBS (WELGENE, Korea), followed by the addition of the concentration of EZ-Cytox enhanced cell viability assay reagent (DoGenBio, Korea), and allowed to react for 1 h. Absorbance values were measured at 450 nm using an Epoch microplate spectrophotometer (BioTek, USA). From the obtained values, cell survival curves were calculated using Excel and prism (v.5.0; GraphPad Software, USA), and IC_50_ values were determined.

### 2.4 Western blot analysis

Proteins were extracted using RIPA buffer (50 mM Tris pH 7.4, 1% NP-40, 150 mM NaCl, 0.25% sodium deoxycholate, 1 mM EDTA, and 1 mM PMSF) supplemented with protease inhibitor cocktail. Protein concentration was determined using the BCA assay (Sigma, St Louis, MO, USA). Proteins were resolved by Tris-Glycine SDS-PAGE and subsequently transferred to a polyvinylidene difluoride (PVDF) membrane. The membrane was incubated with the following antibodies: Caspase-3 (mouse monoclonal, 1:500, #9665, Cell Signaling Technology, USA), Cyclin A2 (mouse monoclonal, 1:500, #4656, Cell Signaling Technology), Cyclin B1 (rabbit polyclonal, 1:500, #4138, Cell Signaling Technology), Cyclin D1 (rabbit monoclonal, 1:500, #2978, Cell Signaling Technology), Cyclin E1 (mouse monoclonal, 1:500, #4129, Cell Signaling Technology, MA, USA),or β-actin (rabbit monoclonal, 1:1000, #4970, Cell Signaling Technology). The membranes were then exposed to X-ray film; protein bands were scanned and measured using ImageJ analysis software (version 1.37; Wayne Rasband, NIH, Bethesda, USA), and normalized by β-actin as the internal control.

### 2.5 Statistical analysis

All experiments consisted of three separate trials. Using the four-parameter logistic analysis (Sigmoidal), IC_50_ values were determined for each essential oil. The western blot data were expressed as the mean ± SEM and analyzed by one-way analysis of variance (ANOVA) followed by Tukey's multiple comparison tests. Statistical analysis was performed by using Graph Pad Prism (version 5.01, GraphPad Software).

## Declaration of Competing Interest

The authors declare that they have no known competing for financial interests or personal relationships that could have appeared to influence the work reported in this paper.

## Appendix Supplementary materials

Image, application 1

Image, application 2
