# Supplementary material for: Data on cytotoxicity of plant essential oils in A549 and Detroit 551 cells
Source: Data Brief. 2020 Aug 16;32:106186. doi: 10.1016/j.dib.2020.106186 (PMC7452582; doi:10.1016/j.dib.2020.106186)

Raw data of Figs and Tables

| A549 cells | | | | | | | | | |
| --- | --- | --- | --- | --- | --- | --- | --- | --- | --- |
| Dendranthema indicum | | | | | | | | | |
| Concentration | Mean | SD | N | Mean | SD | N | Mean | SD | N |
| 0 | 100 | 1.372838 | 6 | 100 | 2.1633 | 3 | 100 | 0.178449 | 3 |
| 0.001 | 96.45753 | 5.311924 | 6 | 94.86211 | 5.357242 | 3 | 98.05296 | 5.848516 | 3 |
| 0.01 | 84.39486 | 7.449005 | 6 | 82.88629 | 8.341036 | 3 | 85.90343 | 7.894235 | 3 |
| 0.1 | 14.42209 | 0.227691 | 6 | 14.28032 | 0.235927 | 3 | 14.56386 | 0.116822 | 3 |
| 1 | 14.53891 | 0.303701 | 6 | 14.28032 | 0.173123 | 3 | 14.79751 | 0 | 3 |
|  |  |  |  |  |  |  |  |  |  |
| Peucedanum japonicum | | | | | | | | | |
| 0 | 100 | 1.394647 | 6 | 100 | 1.836695 | 3 | 100 | 1.220308 | 3 |
| 0.001 | 101.0578 | 1.852514 | 6 | 101.354 | 1.971628 | 3 | 100.7615 | 2.104495 | 3 |
| 0.01 | 88.79544 | 1.197388 | 6 | 88.17204 | 0.248706 | 3 | 89.41884 | 1.535128 | 3 |
| 0.1 | 26.18444 | 3.038036 | 6 | 27.47909 | 3.43441 | 3 | 24.88978 | 2.500115 | 3 |
| 1 | 14.9823 | 0.168271 | 6 | 14.89446 | 0.206936 | 3 | 15.07014 | 0.069421 | 3 |
|  |  |  |  |  |  |  |  |  |  |
| Dendranthema zawadskii | | | | | | | | | |
| 0 | 100 | 9.809448 | 6 | 100 | 12.06664 | 3 | 100 | 9.744705 | 3 |
| 0.001 | 96.63673 | 6.33073 | 6 | 92.02899 | 5.321289 | 3 | 101.2445 | 2.860984 | 3 |
| 0.01 | 93.66903 | 9.327959 | 6 | 91.15179 | 13.42741 | 3 | 96.18627 | 4.268743 | 3 |
| 0.1 | 14.93205 | 0.407274 | 6 | 14.56903 | 0 | 3 | 15.29506 | 0.139065 | 3 |
| 1 | 14.95212 | 0.425845 | 6 | 14.56903 | 0.114416 | 3 | 15.33521 | 0 | 3 |
|  |  |  |  |  |  |  |  |  |  |
| Agastache rugosa | | | | | | | | | |
| 0 | 100 | 1.755574 | 6 | 100 | 2.683021 | 3 | 100 | 0.711687 | 3 |
| 0.001 | 99.3741 | 3.351552 | 6 | 98.79081 | 4.126037 | 3 | 99.95739 | 3.168183 | 3 |
| 0.01 | 98.81954 | 4.950036 | 6 | 95.08263 | 1.743918 | 3 | 102.5565 | 4.040089 | 3 |
| 0.1 | 57.72538 | 6.073695 | 6 | 56.99315 | 8.809676 | 3 | 58.45761 | 3.606324 | 3 |
| 1 | 20.90752 | 2.394999 | 6 | 19.1052 | 1.417041 | 3 | 22.70984 | 1.608401 | 3 |
|  |  |  |  |  |  |  |  |  |  |
| Vitex rotundifolia | | | | | | | | | |
| 0 | 100 | 3.088217 | 6 | 100 | 3.80912 | 3 | 100 | 3.055047 | 3 |
| 0.001 | 95.21641 | 5.257362 | 6 | 96.12078 | 4.9414 | 3 | 94.31204 | 6.49835 | 3 |
| 0.01 | 92.51695 | 4.867914 | 6 | 90.72186 | 2.710325 | 3 | 94.31204 | 6.49835 | 3 |
| 0.1 | 42.36397 | 3.638403 | 6 | 44.17117 | 3.679047 | 3 | 40.55678 | 3.124374 | 3 |
| 1 | 15.40921 | 0.275077 | 6 | 15.45691 | 0.421342 | 3 | 15.36151 | 0.069379 | 3 |
|  |  |  |  |  |  |  |  |  |  |
| Pinus rigida | | | | | | | | | |
| 0 | 100 | 3.088217 | 6 | 100 | 3.80912 | 3 | 100 | 3.055047 | 3 |
| 0.001 | 95.21641 | 5.257362 | 6 | 96.12078 | 4.9414 | 3 | 94.31204 | 6.49835 | 3 |
| 0.01 | 92.51695 | 4.867914 | 6 | 90.72186 | 2.710325 | 3 | 94.31204 | 6.49835 | 3 |
| 0.1 | 42.36397 | 3.638403 | 6 | 44.17117 | 3.679047 | 3 | 40.55678 | 3.124374 | 3 |
| 1 | 15.40921 | 0.275077 | 6 | 15.45691 | 0.421342 | 3 | 15.36151 | 0.069379 | 3 |
|  |  |  |  |  |  |  |  |  |  |
| Orixa japonica | | | | | | | | | |
| 0 | 100 | 3.088217 | 6 | 100 | 3.80912 | 3 | 100 | 3.055047 | 3 |
| 0.001 | 95.21641 | 5.257362 | 6 | 96.12078 | 4.9414 | 3 | 94.31204 | 6.49835 | 3 |
| 0.01 | 92.51695 | 4.867914 | 6 | 90.72186 | 2.710325 | 3 | 94.31204 | 6.49835 | 3 |
| 0.1 | 42.36397 | 3.638403 | 6 | 44.17117 | 3.679047 | 3 | 40.55678 | 3.124374 | 3 |
| 1 | 15.40921 | 0.275077 | 6 | 15.45691 | 0.421342 | 3 | 15.36151 | 0.069379 | 3 |
|  |  |  |  |  |  |  |  |  |  |
| Pinus stobus | | | | | | | | | |
| 0 | 100 | 3.088217 | 6 | 100 | 3.80912 | 3 | 100 | 3.055047 | 3 |
| 0.001 | 95.21641 | 5.257362 | 6 | 96.12078 | 4.9414 | 3 | 94.31204 | 6.49835 | 3 |
| 0.01 | 92.51695 | 4.867914 | 6 | 90.72186 | 2.710325 | 3 | 94.31204 | 6.49835 | 3 |
| 0.1 | 42.36397 | 3.638403 | 6 | 44.17117 | 3.679047 | 3 | 40.55678 | 3.124374 | 3 |
| 1 | 15.40921 | 0.275077 | 6 | 15.45691 | 0.421342 | 3 | 15.36151 | 0.069379 | 3 |
|  |  |  |  |  |  |  |  |  |  |
| Chamaecyparis pisifera | | | | | | | | | |
| 0 | 100 | 3.088217 | 6 | 100 | 3.80912 | 3 | 100 | 3.055047 | 3 |
| 0.001 | 95.21641 | 5.257362 | 6 | 96.12078 | 4.9414 | 3 | 94.31204 | 6.49835 | 3 |
| 0.01 | 92.51695 | 4.867914 | 6 | 90.72186 | 2.710325 | 3 | 94.31204 | 6.49835 | 3 |
| 0.1 | 42.36397 | 3.638403 | 6 | 44.17117 | 3.679047 | 3 | 40.55678 | 3.124374 | 3 |
| 1 | 15.40921 | 0.275077 | 6 | 15.45691 | 0.421342 | 3 | 15.36151 | 0.069379 | 3 |
|  |  |  |  |  |  |  |  |  |  |
| Citrus sunki | | | | | | | | | |
| 0 | 100 | 3.088217 | 6 | 100 | 3.80912 | 3 | 100 | 3.055047 | 3 |
| 0.001 | 95.21641 | 5.257362 | 6 | 96.12078 | 4.9414 | 3 | 94.31204 | 6.49835 | 3 |
| 0.01 | 92.51695 | 4.867914 | 6 | 90.72186 | 2.710325 | 3 | 94.31204 | 6.49835 | 3 |
| 0.1 | 42.36397 | 3.638403 | 6 | 44.17117 | 3.679047 | 3 | 40.55678 | 3.124374 | 3 |
| 1 | 15.40921 | 0.275077 | 6 | 15.45691 | 0.421342 | 3 | 15.36151 | 0.069379 | 3 |

| Detroit 551 cells | | | | | | | | | |
| --- | --- | --- | --- | --- | --- | --- | --- | --- | --- |
| Dendranthema indicum | | | | | | | | | |
| Concentration | Mean | SD | N | Mean | SD | N | Mean | SD | N |
| 0 | 100 | 5.213294 | 6 | 100 | 2.850562 | 3 | 100 | 7.734363 | 3 |
| 0.001 | 99.57408 | 3.148326 | 6 | 98.46659 | 1.757022 | 3 | 100.6816 | 4.244197 | 3 |
| 0.01 | 83.28479 | 4.422926 | 6 | 82.54838 | 5.763905 | 3 | 84.0212 | 3.749154 | 3 |
| 0.1 | 26.02756 | 0.480671 | 6 | 25.73932 | 0.456006 | 3 | 26.31579 | 0.347033 | 3 |
| 1 | 26.39807 | 0.55907 | 6 | 26.17744 | 0.126473 | 3 | 26.61871 | 0.786998 | 3 |
|  |  |  |  |  |  |  |  |  |  |
| Peucedanum japonicum | | | | | | | | | |
| 0 | 100 | 4.107147 | 6 | 100 | 3.666727 | 3 | 100 | 5.359736 | 3 |
| 0.001 | 102.2698 | 5.251016 | 6 | 101.9312 | 5.959769 | 3 | 102.6084 | 5.75067 | 3 |
| 0.01 | 88.79347 | 4.668383 | 6 | 87.17652 | 5.264341 | 3 | 90.41043 | 4.350578 | 3 |
| 0.1 | 29.48204 | 0.999193 | 6 | 28.85284 | 0.354004 | 3 | 30.11124 | 1.087645 | 3 |
| 1 | 26.63606 | 0.343007 | 6 | 26.68984 | 0.463499 | 3 | 26.58228 | 0.265754 | 3 |
|  |  |  |  |  |  |  |  |  |  |
| Dendranthema zawadskii | | | | | | | | | |
| 0 | 100 | 4.123761 | 6 | 100 | 6.367117 | 3 | 100 | 1.404756 | 3 |
| 0.001 | 98.94997 | 3.335427 | 6 | 100.4491 | 3.143713 | 3 | 97.45084 | 3.344782 | 3 |
| 0.01 | 85.32768 | 3.102002 | 6 | 86.7515 | 2.866874 | 3 | 83.90386 | 3.123348 | 3 |
| 0.1 | 27.61581 | 0.444377 | 6 | 27.91916 | 0.449102 | 3 | 27.31245 | 0.126151 | 3 |
| 1 | 27.95163 | 0.671599 | 6 | 28.51796 | 0.343007 | 3 | 27.38529 | 0.2185 | 3 |
|  |  |  |  |  |  |  |  |  |  |
| Agastache rugosa | | | | | | | | | |
| 0 | 100 | 3.707433 | 6 | 100 | 3.721287 | 3 | 100 | 4.529313 | 3 |
| 0.001 | 102.0141 | 8.214758 | 6 | 98.57932 | 2.880551 | 3 | 105.449 | 11.18097 | 3 |
| 0.01 | 96.5159 | 3.717731 | 6 | 97.79006 | 5.338496 | 3 | 95.24175 | 1.088062 | 3 |
| 0.1 | 82.10965 | 5.814538 | 6 | 82.71507 | 3.023002 | 3 | 81.50422 | 8.618815 | 3 |
| 1 | 32.20766 | 2.401944 | 6 | 34.25414 | 1.168007 | 3 | 30.16117 | 0.703388 | 3 |
|  |  |  |  |  |  |  |  |  |  |
| Vitex rotundifolia | | | | | | | | | |
| 0 | 100 | 2.410832 | 6 | 100 | 3.647998 | 3 | 100 | 1.105614 | 3 |
| 0.001 | 100.8927 | 3.053829 | 6 | 99.61876 | 3.144734 | 3 | 102.1666 | 2.925181 | 3 |
| 0.01 | 98.69378 | 2.789963 | 6 | 96.41632 | 1.815613 | 3 | 100.9712 | 0.776414 | 3 |
| 0.1 | 40.56487 | 2.325654 | 6 | 40.44987 | 2.757627 | 3 | 40.67987 | 2.424353 | 3 |
| 1 | 28.64448 | 0.403104 | 6 | 28.93633 | 0 | 3 | 28.35263 | 0.388207 | 3 |
|  |  |  |  |  |  |  |  |  |  |
| Pinus rigida | | | | | | | | | |
| 0 | 100 | 1.488758 | 6 | 100 | 2.250008 | 3 | 100 | 0.691714 | 3 |
| 0.001 | 99.94517 | 5.063968 | 6 | 96.21622 | 1.47124 | 3 | 103.6741 | 4.497734 | 3 |
| 0.01 | 96.81393 | 6.920195 | 6 | 92.50965 | 7.701904 | 3 | 101.1182 | 2.196123 | 3 |
| 0.1 | 26.66973 | 0.844102 | 6 | 26.02317 | 0.353867 | 3 | 27.31629 | 0.633966 | 3 |
| 1 | 26.78954 | 1.036055 | 6 | 26.02317 | 0.482239 | 3 | 27.55591 | 0.830056 | 3 |
|  |  |  |  |  |  |  |  |  |  |
| Orixa japonica | | | | | | | | | |
| 0 | 100 | 9.011704 | 6 | 100 | 12.36199 | 3 | 100 | 7.085775 | 3 |
| 0.001 | 96.95513 | 7.81174 | 6 | 95.86149 | 8.144975 | 3 | 98.04878 | 9.090071 | 3 |
| 0.01 | 93.37907 | 7.021683 | 6 | 93.75 | 10.33274 | 3 | 93.00813 | 4.010199 | 3 |
| 0.1 | 28.66863 | 0.317599 | 6 | 28.80068 | 0.146288 | 3 | 28.53659 | 0.422451 | 3 |
| 1 | 30.25441 | 3.095035 | 6 | 30.99662 | 4.542003 | 3 | 29.5122 | 1.29061 | 3 |
|  |  |  |  |  |  |  |  |  |  |
| Pinus stobus | | | | | | | | | |
| 0 | 100 | 4.726192 | 6 | 100 | 5.525741 | 3 | 100 | 5.030748 | 3 |
| 0.001 | 96.58493 | 4.096002 | 6 | 96.47374 | 3.841309 | 3 | 96.69612 | 5.210598 | 3 |
| 0.01 | 93.47544 | 3.910822 | 6 | 94.48064 | 4.880274 | 3 | 92.47023 | 3.374597 | 3 |
| 0.1 | 28.97173 | 0.325961 | 6 | 28.86163 | 0.459946 | 3 | 29.08183 | 0.133081 | 3 |
| 1 | 29.58579 | 0.237374 | 6 | 29.39824 | 0.132775 | 3 | 29.77334 | 0.133081 | 3 |
|  |  |  |  |  |  |  |  |  |  |
| Chamaecyparis pisifera | | | | | | | | | |
| 0 | 100 | 6.420203 | 6 | 100 | 7.772034 | 3 | 100 | 6.530162 | 3 |
| 0.001 | 98.21803 | 5.960443 | 6 | 100.9535 | 6.584614 | 3 | 95.48255 | 4.797024 | 3 |
| 0.01 | 103.5045 | 8.288215 | 6 | 96.74215 | 4.550064 | 3 | 110.2669 | 3.720658 | 3 |
| 0.1 | 30.78455 | 1.063105 | 6 | 30.23441 | 0.275256 | 3 | 31.3347 | 1.357102 | 3 |
| 1 | 31.06133 | 0.834759 | 6 | 30.87008 | 0.630692 | 3 | 31.25257 | 1.111109 | 3 |
|  |  |  |  |  |  |  |  |  |  |
| Citrus sunki | | | | | | | | | |
| 0 | 100 | 3.976577 | 6 | 100 | 2.794658 | 3 | 100 | 5.6323 | 3 |
| 0.001 | 103.0621 | 5.44104 | 6 | 99.0461 | 2.056044 | 3 | 107.0782 | 4.625924 | 3 |
| 0.01 | 101.5212 | 5.993931 | 6 | 100.0795 | 7.880467 | 3 | 102.963 | 4.634702 | 3 |
| 0.1 | 32.56351 | 1.187129 | 6 | 31.87599 | 0.630942 | 3 | 33.25103 | 1.306544 | 3 |
| 1 | 29.0835 | 0.837198 | 6 | 28.53736 | 0.476948 | 3 | 29.62963 | 0.793716 | 3 |

Figs 3 and 4


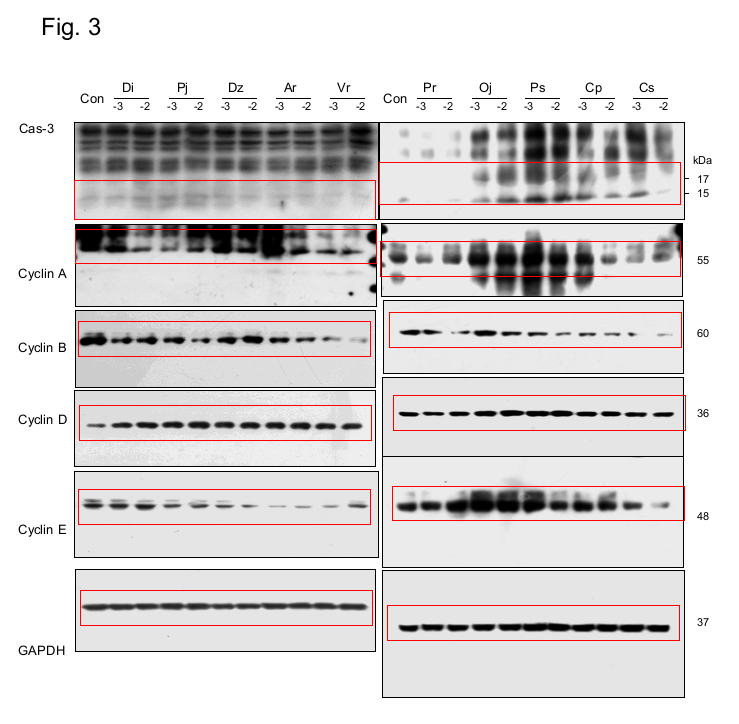


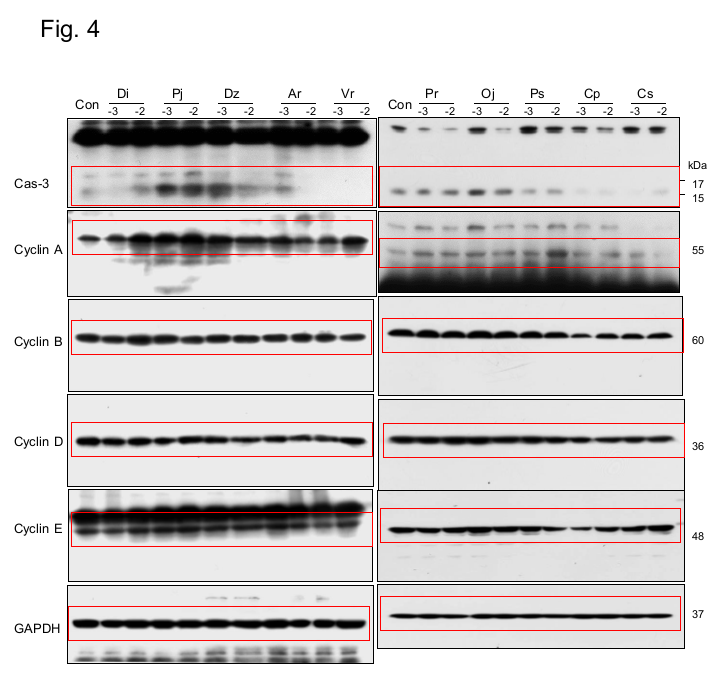

Supplement: Supplementary file 2 [file mmc2.doc]
